# Energy drinks consumption and sleeping quality of students’ universities of the Chiclayo province, Peru

**DOI:** 10.12688/f1000research.177748.1

**Published:** 2026-06-24

**Authors:** María Giulianna Cano Silva, Oliver Llatas Coronado, Sandro Valentino Nole Rojas, Gustavo Adolfo Ventura Seclen

**Affiliations:** 1 Universidad Cesar Vallejo, Trujillo, La Libertad, Peru

**Keywords:** Energy Drinks, Sleep, Life styles, University student.

## Abstract

**Background:**

This study aligns with Sustainable Development Goal 3, Good Health and Well-being, which seeks to promote mental health and reduce premature mortality from non-communicable diseases. The research focuses on stimulant consumption, particularly energy drinks, as a significant risk factor affecting sleep quality among university students. In the local academic context, consuming these beverages has become a widespread and normalized practice. The study aims to address this issue by fostering healthy habits and promoting informed, responsible consumption within the university community. Accordingly, the primary objective is to determine the relationship between energy drink consumption and sleep quality among university students in Chiclayo in 2025.

**Methods:**

A quantitative, non-experimental, cross-sectional design was employed. The population consisted of 12,100 university students in Chiclayo, with a probabilistic sample of 373 participants. Data were collected using two Likert-scale questionnaires, validated by experts and tested for internal reliability.

**Results:**

Findings indicate that energy drink consumption is prevalent among the student population, with most reporting moderate to high intake. Sleep quality was generally acceptable but not optimal, with many participants exhibiting medium to low sleep quality levels. Correlation analyses revealed a strong, negative, inverse relationship between energy drink consumption and sleep quality. Specifically, both the frequency of consumption and the reasons for intake were strongly associated with impaired sleep. Immediate perceived effects after consumption also correlated inversely with restorative sleep, suggesting that short-term energy gains compromise subsequent rest.

**Conclusions:**

The study confirms a strong negative relationship between energy drink consumption and sleep quality among university students in Chiclayo. These findings highlight that stimulant intake constitutes a significant risk factor that undermines restorative sleep, emphasizing the need for interventions promoting healthier consumption habits and improved well-being in the academic community.

## Introduction

Around the world there are some researches that helped to direct the study. As Tomanic et al. (2022), in Serbia, who indicated that energy drink (ED) consumption in adolescents is driven by advertising and widespread accessibility. The main consequence is poor sleep quality, along with psychiatric causes such as severe anxiety, depression, and headaches. In young adults, consumption of EDs containing caffeine, taurine, and glucuronolactone increased platelet aggregation and decreased endothelial function.

On the other hand, Di Martino et al. (2024) in Italy identified that ED consumption among university students is due to the perceived positive effects on mood and stress reduction during the pandemic. The most visible effect was an increase in sleep disorders, directly damaging academic performance. In addition, physical symptoms such as nausea and tachycardia and mental effects such as irritability and anxiety were reported, especially when combining energy drinks with alcohol.

Similarly, Riera-Sampol et al. (2022) comment that in Spain, the high consumption of BE is due to its easy accessibility. University students consume them to alleviate fatigue, increase concentration, and improve academic performance. However, there was an increase in the prevalence of poor sleep quality, which affects concentration and performance.

Furthermore, according to Gonzales et al. (2025), in Colombia, constant advertising campaigns and easy access to stimulants have increased their popularity among university students. As a consequence, they cause acute clinical conditions such as palpitations and insomnia, as well as endocrine, renal, and cardiovascular disorders, including hypertension and arrhythmias.

Nationally, Mendoza et al. (2021), in Lima, indicate that stimulant use is directly related to insomnia symptoms, with a 1.78 times greater risk among users. Reported harmful effects include poor sleep hygiene, headaches, nausea, chest pain, and tachycardia. This highlights that stimulants are a risk factor for optimal rest among the Peruvian university population.

Delving deeper into the Peruvian context, Rodrigo and Rojas (2024) in Huánuco, recorded that stimulant use is employed to prioritize study time or to stay awake. At the same time, the risk of headaches was 2.75 times higher, and sleep quality worsened 3.84 times more. This suggests that sacrificing sleep for academic performance is a widespread practice.

Similarly, Reyes et al. (2024), analyzing universities in Áncash and Lima, indicated that sociodemographic factors, such as age and sex, significantly influence the frequency and reasons for consumption. Among the most common motivations are reducing sleep, using stimulants during exam periods, improving physical performance, and maintaining hydration. High or habitual stimulant intake can cause, in the short term, episodes of stress, anxiety, and insomnia, and in the long term, exacerbate pre-existing conditions such as heart problems or diabetes. In the local area of Chiclayo, high stimulant consumption has been identified among students in academically demanding programs. This intake is associated with the intention of reducing fatigue and extending study time. These stimulants negatively alter the sleep-wake cycle, generating a temporary state of alertness related to sleep disorders and anxiety. This situation aligns with SDG 3: Good Health and Well-being. Therefore, the question arises: What is the relationship between the consumption of energy drinks and sleep quality in university students in Chiclayo, 2025?

The research is theoretically supported by the work of Sandua (2024), who argues that caffeine and stimulants modify the sleep-wake cycle, delaying its onset and reducing the duration and quality of rest. On a practical level, the research contributes by recognizing the link between variables in order to propose actions aimed at promoting responsible habits and consumption within the community. The purpose of this study is to determine the relationship between energy drink consumption and sleep quality among university students in Chiclayo in 2025, showing consumption levels, describing sleep quality, and examining the relationship between its dimensions.

## Theoretical framework

Troya et al. (2024) determined that energy drink consumption among university students was strongly associated with sex, with 77% of consumption occurring in men. It was concluded that academic pressure and stress are significant and relevant psychological factors that particularly encourage students with higher levels of effort to consume these drinks to cope with the demands of their academic training.

In the same year, Bendini et al. (2024), in a study focused on medical students, found that 65.8% presented poor sleep quality and 67.7% abnormal daytime sleepiness. It was also observed that students who consumed energy drinks had greater difficulty falling asleep, in contrast to those who did not consume them and slept longer at night.

In line with this, Cala et al. (2023) established a significant association between energy drinks consumption and insomnia, with a prevalence of 43.9% among postgraduate health students. The study concluded that BE consumption increases the risk of insomnia by 1.7 times.

Similarly, Bressan et al. (2024), in a study investigating the prevalence of alcohol consumption among medical students, found that 74.12% of this population consumed alcohol, with the main occasions of consumption being before or during exam weeks (67.5%), at parties (63.2%), and during daily study sessions (55.6%). 64.5% reported side effects, and 74.6% continued consuming these beverages despite knowing their adverse effects. Therefore, it was concluded that alcohol is considered an option to enhance academic performance.

In turn, Espinosa et al. (2025) determined a direct correlation between alcohol consumption and the deterioration of health and academic performance, concluding that these findings are relevant for the implementation of institutional programs focused on reducing the consumption of such beverages.

Similarly, Kaldenbach et al. (2024) showed that BE consumption was notably associated with negative sleep indicators. Daily consumption was linked to a sleep duration of less than six hours, greater initial difficulty falling asleep, and difficulty falling back asleep after achieving sleep.

By the way, Riera et al. (2022) found that caffeine consumption, including energy drinks, is a crucial cofactor for the negative self-perception of sleep quality and rest among university students; they also identified that energy drinks are primarily consumed by men.

In an opposite way, Rosado et al. (2025) did not find a direct and general relationship between energy drink consumption and the perceived health of their respondents. Based on these results, they concluded that addressing the issue of energy drink consumption among the university population requires considering the students' context.

On the other hand, the following hypothesis was proposed: there is a relationship between the consumption of energy drinks and sleep quality in university students in the city of Chiclayo in 2025.

### Energy drink consumption

Definition. According to Ankita Chatterjee (2019), this variable is defined as the combination of stimulants and energy enhancers designed to improve physical and cognitive performance, whose main active component is caffeine.

### Dimensions

The dimensions covered by energy drink consumption are:

- Consumption
- Motives
- Perceived effects

We have taken these dimensions in our research, as we consider them necessary for the application of our instrument.

### Sleep quality

Definition. It is defined according to Ogundele et al. (2023, cited in Wang, 2024) as the ability to fall asleep and maintaining sleep uninterruptedly, along with the facility to maintain daytime functional performance without impairment or discomfort; poor sleep quality leads to difficulties in concentration, learning, behavioral changes, and an increased risk of developing illnesses.

### Dimensions

Three crucial aspects stand out in determining the dimensions based on sleeping quality:

- Sleep duration
- Sleep efficiency
- Daytime consequences

Similarly, we have included these variables because they are relevant to the measurement of the variable through the application of the instrument.

## Methodology

### Type, design, and scope

According to Baena (2017), this research employed a basic research approach, as it focuses on discovering general principles theoretically rather than seeking immediate practical results. Instead, it aims to expand theoretical knowledge. Therefore, applying this type of research was the most appropriate, since we sought to understand the relationship between energy drink consumption and sleep quality theoretically, rather than to seek or propose solutions or improvements.

Likewise, a quantitative approach was used, which, according to Hernandez y Mendoza (2018), is a structured, sequential, and systematic process that objectively measures variables using statistical data collection techniques. The data can be interpreted numerically to test hypotheses and identify causal relationships that confirm the studied problem aligns with objective reality.

Finally, based on Arias and Covinos' (2021) definition, the design used was non-experimental and cross-sectional, since there were no changes or modifications to the study variables and, furthermore, because the data were collected only once and at a specific point in time, and then described and analyzed. Thanks to this approach, it was possible to observe and collect information about the event under study, obtaining relevant data without altering the variables, thus ensuring the impartiality of the results.

### Population and sample

For this research, the population, according to Baena (2017), comprises the total group of people or units with one or more similar characteristics, from whom information can be extracted, thus forming the study universe. Arias and Covinos (2021) add that this set of units will be tested through observation, measurement, or surveys.

In the present study, the population consisted of 12,100 university students in Chiclayo (INEI, 2023), to whom the following inclusion criteria were applied: individuals between 18 and 34 years of age who reside in or study in the city of Chiclayo. Likewise, the exclusion criteria include individuals who have not given their informed consent to participate in the study and those who suffer from a chronic or oncological disease that prevents their adequate participation.

The sample, according to Gómez (2006), is a significant part of the study elements or population. For the selection of the probabilistic sample, the sample size calculator adjusted to 95% confidence was used, thus obtaining the sample quantity of 373 university students in Chiclayo, 2025.

### Procedure

The procedure for undertaking this study is structured into several crucial stages that enable the understanding of the phenomenon within its context. The process initiates with the identification of a general idea, followed by the clear formulation of the problem to be addressed. Subsequently, the methodology is designed, appropriate samples are selected, the information is collected and processed, and ultimately, the results obtained are interpreted and analyzed to yield meaningful conclusions.

### Techniques and instruments for data collection

Depending on the case and type of study, Hernandez et al. (2018) emphasize the importance of a good selection of data collection methods and tools, ranging from interviews and direct observation to surveys, etc.

For this research, the survey was used as the primary data collection technique, based on the criteria provided by Santisteban (2014), who indicates that this technique, focused on formulating a series of organized questions for a particular group of individuals, aims to gather relevant information on a specific topic. This conceptualization allowed us to choose this method because the questions provided critical information for the study, thus aiding in the analysis of the problem.

Regarding the data collection instrument, the questionnaire was chosen. As Santisteban (2014) again states, this is a tool that the researcher designs using a series of organized and specific questions, with which they can extract very specific information from a predetermined population sample. For this study, the instrument consisted of two structured questionnaires, each one with a total of 15 questions organized into three dimensions per variable. Responses were arranged to be measured on a Likert scale from 1 to 5, where 1 represents “never” and 5 represents “always.”

Regarding the observation about the evaluation tool used, it is stated that the research instrument is original work and was validated by experts in the field. Therefore, obtaining a third-party copyright license is not applicable.

### Validity and reliability

The technique used was the survey and as instruments applied were two questionnaires, both of them validated by three experts in the field, they were PhD. Carlos Francisco Cadenillas Barturen, Mg. Victor Humberto Barboza Rojas and Mg. Deyssy Soledad Medina Gamonal. The questionnaires' reliability was obtained based on Cronbach' s alpha, the first questionnaire was 0.992 and the second, 0.741; which can be seen in Tables 7 and 8, respectively.

**Table 1.** Level of Energy drinks consumption.

		Frequency	Percentage
Valid	Low	49	13,1
	Medium	225	60,3
	High	99	26,5
	Total	373	100,0

Note: Based on the results obtained from Table 1 for the first variable, it is estimated that 60.3% of university students have a medium level of energy drink consumption, while 26.5% have a high level. The proportion of students with a low level of consumption is small, at 13.1%.

**Table 2.** Level of Sleeping qualitiy.

		Frequency	Percentage
Valid	Low	120	32,2
	Medium	213	57,1
	High	40	10,7
	Total	373	100,0

Note: Considering the results shown in Table 2 for the second variable, it was observed that a higher concentration of students consider themselves to have a medium and low level of sleep quality, 57.1% and 32.2%, respectively.

**Table 3.** Relationship of dimension 1 of energy drinks consumption with sleeping quality.

Correlations			
		*Consumption*	Sleeping quality
*Consumption*	Correlation coefficient	1	-,770
	Sig. (bilateral)	.	,0
	N°	373	373
Sleeping quality	Correlation coefficient	-,770	1
	Sig. (bilateral)	,0	.
	N°	373	373

Note: Thus, in Table 3, it can be inferred that there is a relationship between dimension 1 of the energy drink consumption variable and the sleep quality variable. This is because a bilateral significance of less than 0.000 < 0.005 is observed, placing it in a very strong negative category (r = -0.770). This indicates that high consumption of energy drinks has a negative influence on sleeping quality.

**Table 4.** Relationship of dimension 2 of energy drinks consumption with sleeping quality.

Correlations			
		*Motives*	Sleeping quality
*Motives*	Correlation coefficient	1	-,748
	Sig. (bilateral)	.	,0
	N°	373	373
Sleeping quality	Correlation coefficient	-,748	1
	Sig. (bilateral)	,0	.
	N°	373	373

Note: In Table 4, a correlation was found between dimension 2 of the energy drink consumption variable and the sleep quality variable. A bilateral significance of less than 0.000 < 0.005 was observed, placing it in a considerably negative category (r = -0.748). The more reasons students have to consume energy drinks, the lower their sleep quality levels will be.

**Table 5.** Relationship of dimension 3 of energy drinks consumption with sleeping quality.

Correlations			
		*Perceived effects*	Sleeping quality
*Perceived effects*	Correlation coefficient	1	-,762
	Sig. (bilateral)	.	,0
	N°	373	373
Sleeping quality	Correlation coefficient	-,762	1
	Sig. (bilateral)	,0	.
	N°	373	373

Note: In Table 5, it was possible to deduce that there is a correlation between dimension 3 of the energy drink consumption variable and the sleep quality variable, as a result of obtaining a bilateral significance of less than 0.000 < 0.005, classified as a very strong negative level (r = -0.762). This relationship may explain that the greater the perceived effects of ED consumption are, the less easy it is for them to enjoy quality sleep.

**Table 6.** Relationship between energy drinks consumption with sleeping quality.

Correlations			
		*Energy drinks consumption*	Sleeping quality
*Energy drinks consumption*	Correlation coefficient	1	-,775
	Sig. (bilateral)	.	,0
	N°	125	373
Sleeping quality	Correlation coefficient	-,775	1
	Sig. (bilateral)	,0	.
	N°	373	373

Note: In Table 6, based on the responses of the 373 respondents, it was determined that there is a correlation between energy drink consumption and sleep quality among university students in the province of Chiclayo, and that poor sleeping quality is a consequence of high ED consumption, due to a bilateral significance of less than 0.000 < 0.005, with the result being very strongly negative (r = -0.775).

**Table 7.** Variable reliability statistics: 1.

Cronbach's alpha	Number of elements
,992	15

**Table 8.** Variable reliability statistics: 2.

Cronbach's alpha	Number of elements
,741	15

About the treatment of the collected data, descriptive statistics worked to evaluate the levels of each variable as important evidence for the first two specific objectives. Also, inferential statistics were used to find correlations between the variables and dimensions. This, because the data was not normally distributed as Table 9 showed. Additionally, subgroup analyses were performed by analyzing the correlations between the dimensions of energy drink consumption and the dimensions of sleep quality. Everything was possible by processing the data in excel and SPSS version 26.

**Table 9.** Normality tests.

Correlations			
	Statistical	gl	Sig.
*Energy drinks consumptions*	,111	373	,000
Sleeping quality	,094	373	,000

### Data analysis

First, in order to analyze and interpret the information, it was necessary to process and transform the data obtained. The study focused on determining whether there is a relationship between energy drinks consumption and sleep quality among university students in Chiclayo. To this end, clear and precise objectives were set, which allowed for a systematic and effective approach, ensuring that the conclusions obtained were clear and well-supported.

Second, to collect the data, a sample of 373 university students in Chiclayo was surveyed, ensuring that they’d satisfy the inclusion and exclusion criteria. During this crucial stage, each form collected was carefully and rigorously reviewed to ensure that it was complete, guaranteeing the reliability of the information, the accuracy of the results, and a solid analysis of the problem.

Third, the questionnaires were designed on a Likert scale, which allowed for a structured and orderly measurement of responses. Once collected, they were organized and numbered in Excel. After being loaded into the IBM SPSS Statistics V.26 program, they were processed after obtaining a highly reliable Cronbach's alpha coefficient.

Finally, the results were adapted to the APA format, ensuring that all statistical analyses were explained correctly and provided valuable information for the study.

### Ethical criterial

A total of 373 participants from the university population were included in the study. The process began with the receipt of written informed consent, after providing a comprehensive explanation of the objectives and methodology of the research. Data collection was carried out through surveys and questionnaires. The study was based on adherence to sound ethical principles, ensuring intellectual honesty in the handling, safeguarding, and proper use of the information collected.

The research was conducted in strict accordance with the Declaration of Helsinki and current national legislation. Study subjects were provided with a document specifying the procedures, benefits, and confidentiality guarantees. Likewise, the correct attribution of third-party ideas was ensured through formal citation, guaranteeing respect for intellectual property, and objectivity was maintained in the academic approach employed.

The requirement for participation was the formal, signed consent of each student. The study was carried out with the approval of the Institutional Research Ethics Committee of César Vallejo University, under approval code RCU No. 0659-2024-UCV. Given the nature of the methodology, the committee considered formal consent to be essential, without granting any exemptions to the process.

Once the right of university students to withdraw from the study at any time, without experiencing negative consequences, was confirmed. Just then to protect the identity of the subjects, the responses were coded, replacing names with codes. Finally, it is emphasized that the research did not involve physical risks, experimental procedures, or medical interventions that could endanger the well-being of the participants.

## Results

## Discussion - Conclusions - Recommendations

In line with the general objective, it is concluded that there is a strong, negative, inverse association between energy drink consumption and sleeping quality in university students in Chiclayo. This result implies that, as energy drink consumption increases, sleeping quality tends to decline, suggesting that consumption of these stimulants is a significant risk factor with a negative impact. These findings are similar to the results of studies conducted in other contexts, such as Kaldenbach et al. (2024), who found a negative and high relationship between the variables in Noruega. This suggests that the problem may be present regardless of socioeconomic status or quality of life. It is recommended that local health center directors be involved in the design and implementation of preventive measures aimed at reducing energy drinks consumption among the Chiclayo university’s population, as well as promoting healthy rest and sleep hygiene practices, thereby strengthening local public health.

Regarding specific objective 1, a marked pattern of moderate to high consumption of energy drinks was identified in the vast majority of university students, with more than half of those surveyed reporting medium or high levels of consumption. This predominant behavior suggests that the consumption of these products has become a widespread and normalized practice in the local university academic context, possibly as a recurring strategy to perform well in the face of academic demands. This practice reflects a disconnect between high consumption and health. This analysis connects with Troya et al. (2024) study, who in Venezuela, discovered that many university students by drinking these stimulants find a way out of stress and academic pressure. Therefore, it is recommended that, through education and prevention strategies led by local university administrators, the purchase and sale of these beverages within the university be avoided or restricted to mitigate the potential risks associated with this behavior on university health.

In addition to the above, in relation to specific objective 2, it was observed that the quality of sleep among university students was predominantly average to low. This situation is concerning, as it indicates that a large part of the university population experiences insufficient sleep quality, probably due to the academic workload characteristic of this stage of education and unhealthy lifestyles. This finding may be attached to Bendini et al. (2024) study, who established that poor sleeping quality among Brazilian students was caused by their energy drinks consumption. This can be taken as an indicative of a public health problem that could affect the well-being, performance, and long-term health of this population sector. In view of this, it is urgently recommended that the Peruvian Minister of Health become involved in the development of sleep hygiene policies and ensure the provision of special rest areas or zones in higher education institutions, highlighting the importance of nighttime rest as a fundamental component of the overall health of the university community.

Finally, with respect to specific objective 3, an inverse, negative, and significant relationship was identified between the three dimensions of energy drink consumption and sleep quality. The correlation found was very strong for the consumption dimension, which shows that the regularity and daily nature with which students have assimilated this practice constitutes a relevant risk factor. The reasons for consumption suggest that the underlying causes are related to a need for greater energy performance, creating a vicious circle of dependence. This interpretation is clearly reflected with Cala et al. (2023) study, who conclude that constant consumption of these drinks could go further worse on the Colombian students’ sleep quality, who just want to get a sense of control on their grades even if they damage their health in the path. Likewise, the perceived effects indicate that, despite students being aware of the adverse effects such as insomnia and anxiety, they continue to engage in this practice. It is recommended that the Minister of Education address this issue comprehensively, focusing on preventing the consumption of energy drinks and raising awareness among the university population that the perceived effects of long-term consumption lead to a decline in academic performance and aggravate health problems.

## Ethics and consent

The research was conducted with a sample of 373 students selected from the target university population. The process began with the request and receipt of written informed consent. Prior to this, each participant was given a detailed explanation of the objectives, methodology, and procedures involved in the study. This work is based on respect for sound ethical principles, ensuring intellectual honesty in the collection and truthful use of the data obtained.

The study was conducted in accordance with the ethical principles enshrined in the Declaration of Helsinki and relevant national regulations. Study subjects were provided with clear instructions on the purpose of the research. Likewise, their right to withdraw from participation at any time and without negative consequences was guaranteed. The confidentiality of the information provided was maintained throughout the development and subsequent conclusion of the work.

All individuals gave their informed consent before beginning their collaboration. To this end, they were given a document detailing the objectives, procedures, benefits, possible risks, and confidentiality assurances. The research was conducted in compliance with the approval granted by the Institutional Ethics Committee of César Vallejo University. This committee determined that no exemption from the consent process was appropriate, as it was essential for each participant to express their agreement explicitly and formally, given that the methodology used was a questionnaire.

To ensure the confidentiality of the information, responses were coded instead of using personal names, thus eliminating any potential for individual identification of respondents. Additionally, it is reaffirmed that the study does not include medical interventions or experimental procedures that could compromise the safety or well-being of the students. In this way, the protection and care of the subjects involved was ensured in accordance with established guidelines.

Furthermore, participants were assured of confidentiality that protected their identities and provided them with the right to withdraw their participation at any time without facing negative consequences. Risks associated with personal and sensitive data were mitigated through the use of coded responses.

## Data Availability

Zenodo: Energy drinks consumption and sleeping quality of students' universities of the Chiclayo province, Peru. https://doi.org/10.5281/zenodo.18262708 ( Nole et al., 2026). This article contains the following underlying data:
• Energy drinks consumption.xlsx (Dataset containing survey responses regarding energy drink intake among university students). • Sleeping quality.xlsx (Dataset containing survey responses regarding sleep quality metrics). The data are available under the terms of the Creative Commons Attribution 4.0 International license (CC-BY 4.0). Zenodo: Energy drinks consumption and sleeping quality of students' universities of the Chiclayo province, Peru. https://doi.org/10.5281/zenodo.18262708 ( Nole et al., 2026). This project includes the following extended data:
•
Research_Instrument_1000.pdf (Questionnaires created and validated by experts to mesure study variables). • Informed consent.pdf (Template of the written informed consent provided to and signed by participants). The data are available under the terms of the Creative Commons Attribution 4.0 International license (CC-BY 4.0). 1 César Vallejo University, Carretera Pimentel Km. 3.5 Cano Silva María Giulianna ^1^ Roles: Conceptualization, Research, Methodology. Nole Rojas Sandro Valentino ^1^ Roles: Software, Data curation, Formal analysis, Validation Llatas Coronado Oliver ^1^ Roles: Writing and Editing, Conceptualization. Ventura Seclén Gustavo Adolfo ^1^ Roles: Software, Data curation, Writing – Review and Editing, Conceptualization, Methodology, Writing – Preparation of the original draft, Supervision Zenodo: STROBE checklist for Energy drinks consumption and sleeping quality of students' universities of the Chiclayo province, Peru. https://doi.org/10.5281/zenodo.18262708 ( Nole et al., 2026). The data are available under the terms of the Creative Commons Attribution 4.0 International license (CC-BY 4.0).
