# Supplementary material for: Energy drinks consumption and sleeping quality of students’ universities of the Chiclayo province, Peru
Source: F1000Res. 2026 Jun 24;15:1007. [Version 1] doi: 10.12688/f1000research.177748.1 (PMC13653107; doi:10.12688/f1000research.177748.1)
Supplement: Peer review history

# Reviewer report: Reviewer response for version 1

**DOI:** 10.5256/f1000research.196024.r526443

**Published:** 2026-10-09

**Authors:** Lindon Vela Meléndez

**Affiliations:** 1 Universidad Nacional Pedro Ruiz Gallo, Lambayeque, Lambayeque, Peru

The work is generally relevant; however, some data need to be clarified and corrected:

 Please indicate whether the results are included in another section of the manuscript or are missing from it.

 The note for Table 5: The threshold “0.000 < 0.005” is incorrect; the conventional level is 0.05, and p is never exactly zero, so it should be written as p < 0.001.

 Table 6 reports N = 125 for consumption and 373 for sleep. If there were 125 valid cases, the analysis would not correspond to the stated sample.

 I approve this manuscript with reservations.

Is the work clearly and accurately presented and does it cite the current literature?

Yes

If applicable, is the statistical analysis and its interpretation appropriate?

Partly

Are all the source data underlying the results available to ensure full reproducibility?

Yes

Is the study design appropriate and is the work technically sound?

Partly

Are the conclusions drawn adequately supported by the results?

Partly

Are sufficient details of methods and analysis provided to allow replication by others?

Yes

Reviewer Expertise:

Economy, education

I confirm that I have read this submission and believe that I have an appropriate level of expertise to confirm that it is of an acceptable scientific standard, however I have significant reservations, as outlined above.

# Reviewer report: Reviewer response for version 1

**DOI:** 10.5256/f1000research.196024.r526440

**Published:** 2026-10-09

**Authors:** Lilian Roxana Paredes López

**Affiliations:** 1 Pedro Ruiz Gallo National University, Lambayeque, Peru

1. Is the work clearly and accurately presented and does it cite current literature?

 Yes. The manuscript clearly presents the research problem and incorporates recent literature concerning energy drink consumption and sleep quality. The introduction provides appropriate international, national, and local context. Minor improvements could further strengthen the synthesis of the evidence and clarify the distinction between energy drinks, stimulants, caffeine, and alcoholic beverages.

 2. Is the study design appropriate and technically sound?

 Yes. The quantitative, non-experimental, cross-sectional design is appropriate for examining the relationship between energy drink consumption and sleep quality. The study clearly identifies its population, sample, and inclusion and exclusion criteria.

 3. Are sufficient methodological details provided for replication?

 Yes. The manuscript describes the survey technique, questionnaires, Likert scale, expert validation, and reliability assessment. Cronbach’s alpha coefficients of 0.992 and 0.741 indicate adequate internal consistency.

 4. Is the statistical analysis and interpretation appropriate?

 Yes. Spearman’s correlation is appropriate considering the reported non-normal distribution of the data. The statistical procedures were conducted using Excel and SPSS version 26.

 5. Are the source data available for reproducibility?

 The manuscript provides relevant methodological information and describes data processing procedures. A more explicit Data Availability Statement would further strengthen reproducibility.

 6. Are the conclusions adequately supported by the results?

 Yes. The findings support an inverse association between energy drink consumption and sleep quality. The conclusions are generally supported, although causal wording should be avoided because of the cross-sectional design.

 Overall assessment: Favorable.

Is the work clearly and accurately presented and does it cite the current literature?

Yes

If applicable, is the statistical analysis and its interpretation appropriate?

Yes

Are all the source data underlying the results available to ensure full reproducibility?

Yes

Is the study design appropriate and is the work technically sound?

Yes

Are the conclusions drawn adequately supported by the results?

Yes

Are sufficient details of methods and analysis provided to allow replication by others?

Yes

Reviewer Expertise:

University educationSocial sciencesData statistics

I confirm that I have read this submission and believe that I have an appropriate level of expertise to confirm that it is of an acceptable scientific standard.

# Reviewer report: Reviewer response for version 1

**DOI:** 10.5256/f1000research.196024.r526447

**Published:** 2026-10-07

**Authors:** Yefferson Llonto-Caicedo

**Affiliations:** 1 Universidad Nacional Pedro Ruiz Gallo, Lambayeque, Peru

Sampling and representativeness

 The article describes the sample as a probability sample but does not explain how participants were actually selected. It should identify the universities included, the sampling frame, the random selection procedure, the distribution of participants across institutions, and how refusals or replacements were handled. If participants were recruited through convenience sampling or an open survey link, the actual procedure should be described, and the scope of generalization should be adjusted accordingly. Obtaining 373 responses does not, in itself, demonstrate that probability sampling was used.

 The geographical scope should also be consistent: the title refers to the province, whereas the eligibility criteria and objectives also refer to the city of Chiclayo. The stated population of 12,100 students requires a traceable source, including the original table or record, and an explanation of how this figure corresponds to the population studied in 2025.

 Sample size justification 

 Only the confidence level and final sample size are reported. The formula, its parameters, the sources supporting the assumptions, any finite population correction applied, and the allowance for nonresponse should be provided.

 The response to the reviewer should distinguish between two situations: if the sample size was calculated to estimate a proportion, the desired precision and expected proportion should be reported; if it was calculated to detect a correlation, the anticipated correlation coefficient, significance level, and statistical power should be specified. A calculation must not be retrospectively reconstructed and presented as the original procedure.

Is the work clearly and accurately presented and does it cite the current literature?

Yes

If applicable, is the statistical analysis and its interpretation appropriate?

Yes

Are all the source data underlying the results available to ensure full reproducibility?

Yes

Is the study design appropriate and is the work technically sound?

Partly

Are the conclusions drawn adequately supported by the results?

Yes

Are sufficient details of methods and analysis provided to allow replication by others?

Yes

Reviewer Expertise:

Economia y agroexportación

I confirm that I have read this submission and believe that I have an appropriate level of expertise to confirm that it is of an acceptable scientific standard, however I have significant reservations, as outlined above.

# Reviewer report: Reviewer response for version 1

**DOI:** 10.5256/f1000research.196024.r520193

**Published:** 2026-09-28

**Authors:** Pei Nee Chong

**Affiliations:** 1 Nutrition, Dietetic and Food S, International Medical University School of Health Sciences, Kuala Lumpur, Federal Territory of Kuala Lumpur, Malaysia

This manuscript examines the association between energy drink consumption and sleep quality among university students in Chiclayo, Peru. The topic is relevant and the sample size of 373 participants is potentially adequate. However, substantial revisions are required before the manuscript is scientifically sound. The main concerns relate to the literature review, sampling and sample-size justification, questionnaire validity, statistical reporting, and overinterpretation of the findings.

 1. Is the work clearly and accurately presented and does it cite the current literature?

 The Introduction is overly descriptive and presents studies individually rather than synthesizing the evidence. Several concepts are repeated, and energy drinks, stimulants, caffeine, and alcoholic beverages are sometimes treated interchangeably. The authors should clearly define the exposure and focus the literature review on evidence directly relevant to energy drinks and sleep quality.

 Several statements also appear causal, such as energy drinks “causing” poor sleep or being a “risk factor.” These should be revised to reflect the observational nature of the cited evidence. The literature should be updated and the specific claims should be checked against the original cited studies.

 Required: Restructure and condense the Introduction, improve synthesis of the literature, clarify terminology, verify citations, and avoid unsupported causal claims.

 2. Is the study design appropriate and is the work technically sound?

 A cross-sectional, non-experimental design is appropriate for examining an association between energy drink consumption and sleep quality. However, it cannot establish causality.

 The sampling procedure is insufficiently described. Although the authors describe the sample as probabilistic, they do not explain how the 373 students were actually selected. The sample-size calculation is also inadequately reported; only a 95% confidence level and the final sample size are provided.

 Required: Clearly describe the sampling method and provide the complete sample-size calculation, including the formula, primary outcome, assumed effect size/prevalence, α, power, and source of the assumptions. The authors should also acknowledge the limitations of a cross-sectional design.

 3. Are sufficient details of methods and analysis provided to allow replication by others?

 The Methods section contains lengthy textbook definitions but insufficient details about the actual study procedures. The development/source of the two questionnaires, scoring procedures, validity assessment, recruitment process, data collection, and handling of missing data are not adequately described.

 The authors report validation by three experts and Cronbach's α values of 0.992 and 0.741, but provide insufficient information about the validation process and which coefficient corresponds to each questionnaire.

 Required: Provide clear information on questionnaire development/adaptation, validity, reliability, scoring, participant recruitment, data collection, and data management. Remove unnecessary textbook definitions.

 4. Is the statistical analysis and its interpretation appropriate?

 Spearman's correlation appears appropriate if the variables were ordinal/non-normally distributed, but the statistical reporting requires improvement. The authors should explicitly identify the test, report Spearman's ρ, p-values, and sample size, and avoid reproducing SPSS correlation matrices.

 The reported correlations are very strong (approximately ρ = −0.75 to −0.78) and should be verified, particularly given the apparent use of author-developed Likert questionnaires.

 There is also an apparent discrepancy in Table 6, where N = 125 appears for energy-drink consumption while N = 373 is reported for sleep quality. This must be clarified.

 Most importantly, the authors interpret the correlations causally, using terms such as “consequence,” “influence,” and “risk factor.” A cross-sectional correlation cannot establish causality.

 Required: Verify the analyses and sample sizes, improve statistical reporting, clarify scoring direction, and revise all causal interpretations.

 5. Are all the source data underlying the results available to ensure full reproducibility?

 The manuscript does not provide a clear Data Availability Statement or indicate whether the underlying anonymized data and analysis materials are available.

 Required: Provide a Data Availability Statement and, where ethically permissible, provide access to de-identified data, questionnaires, coding/scoring information, and analysis syntax or equivalent documentation.

 6. Are the conclusions adequately supported by the results?

 The results support a strong inverse association between energy drink consumption and sleep quality, but they do not establish that energy drink consumption causes poor sleep. The current conclusion overstates the evidence by describing energy drink consumption as a risk factor and poor sleep as a consequence.

 The conclusion should instead state that higher energy drink consumption was associated with poorer sleep quality, while acknowledging that the cross-sectional design cannot determine temporal direction or causality.

 Required: Revise the Results, Discussion, and Conclusion to distinguish association from causation and acknowledge potential reverse causality and confounding.

 Please view the additional review comments in the file found **here** .

Is the work clearly and accurately presented and does it cite the current literature?

No

If applicable, is the statistical analysis and its interpretation appropriate?

No

Are all the source data underlying the results available to ensure full reproducibility?

No

Is the study design appropriate and is the work technically sound?

No

Are the conclusions drawn adequately supported by the results?

No

Are sufficient details of methods and analysis provided to allow replication by others?

No

Reviewer Expertise:

nutraceutical product, nutrigenomic, pediatric obesity, metabolic health

I confirm that I have read this submission and believe that I have an appropriate level of expertise to state that I do not consider it to be of an acceptable scientific standard, for reasons outlined above.
